# Identification of a novel de novo mutation of *SETBP1* and new findings of *SETBP1* in tumorgenesis

**DOI:** 10.1186/s13023-023-02705-6

**Published:** 2023-05-07

**Authors:** Hongdan Wang, Yue Gao, Litao Qin, Mengting Zhang, Weili Shi, Zhanqi Feng, Liangjie Guo, Bofeng Zhu, Shixiu Liao

**Affiliations:** 1 Medical Genetic Institute of Henan Province, Henan Provincial People’s Hospital, Zhengzhou University People’s Hospital, Zhengzhou, China; 2 National Health Commission Key Laboratory of Birth Defects Prevention, Henan Key Laboratory of Population Defects Prevention, Zhengzhou, China; 3 Department of Urology, The First People’s Hospital of Zhengzhou, Zhengzhou, China; 4 Guangzhou Key Laboratory of Forensic Multi-Omics for Precision Identification, School of Forensic Medicine, Southern Medical University, Guangzhou, China

**Keywords:** *SETBP1*, SETBP1 haploinsufficiency disorder, Schinzel-Giedon syndrome, Tumorgenesis

## Abstract

**Background:**

In the past decade, *SETBP1* has attracted a lot of interest on that the same gene with different type or level (germline or somatic) of variants could provoke different pathologic consequences such as Schinzel-Giedon syndrome, SETBP1 Haploinsufficiency Disorder (SETBP1-HD) and myeloid malignancies. Whole exome sequencing was conducted to detect the etiology of a pregnant woman with mental retardation. As a new oncogene and potential marker of myeloid malignancies, somatic *SETBP1* variants in other cancers were rarely studied. We performed a pan-cancer analysis of *SETBP1* gene in different cancers for the first time.

**Results:**

A novel heterozygous mutation of the *SETBP1* gene (c.1724_1727del, p.D575Vfs*4) was found in the patient and the fetus and the mutation was predicted to result in a truncated protein. Reduced *SETBP1* expression was associated with SETBP1-HD. The pan-cancer analysis of *SETBP1* showed that *SETBP1* overexpression should be given special attention in Bladder Urothelial Carcinoma (BLCA) and Stomach adenocarcinoma (STAD).

**Conclusions:**

The de novo *SETBP1* mutation was the genetic cause of SETBP1-HD in the family. BLCA and STAD might be related to *SETBP1* overexpression.

**Supplementary Information:**

The online version contains supplementary material available at 10.1186/s13023-023-02705-6.

## Introduction

SET-binding protein 1 (*SETBP1*, OMIM611060) localized in chromosome 18q21 and expressed predominantly in the nucleus [1]. This gene encoded a 170 kDa protein, and many transcriptional variants encoding different isoforms have been found (provided by RefSeq, Aug 2011). The known and predicted protein domains of SETBP1 contained a SKI homology region, a SET binding domain, three AT hooks and a repeat domain [2]. SETBP1 binded the SET nuclear oncogene which was involved in DNA replication by its SET binding domain [1]. Recent studies demonstrated the involvement of *SETBP1* germ line mutations in Schinzel-Giedion syndrome (SGS, OMIM269150) and SETBP1 Haploinsufficiency Disorder (SETBP1-HD, OMIM616078) and *SETBP1* somatic mutations in several hematological malignancies [3–6].

In 1978, SGS was first described in two siblings and was a highly recognizable disease [7]. SGS was a rare and severe multi-system disorder characterized by recognizable facial features (prominent forehead, midface retraction and short nose), severe intellectual disability, multiple congenital malformations (cardiac defects, genitourinary and kidney malformations, and skeletal abnormalities). Some SGS patients could be found to have other nervous system abnormalities such as deafness and intractable epilepsy [2, 6–8]. Most individuals of SGS did not live beyond 10 years. All reported *SETBP1* mutations in previous individuals of SGS were located exclusively in the SKI homologous region of exon 4 (codons 868–871) [2, 5]. Mutations in these hotspots disrupted a degron for the protein degradation, which resulted in the accumulation of SETBP1 protein. The pathogenic mechanism in SGS was considered to be the gain of function or dominant-negative effects [2, 5].

SETBP1-HD with a much less severe phenotype than SGS, also known as the Autosomal Dominant Mental Retardation type 29 (MRD29), was characterized by broad clinical spectrum, such as mild motor developmental delay, hypotonia, intellectual abilities ranging from normal to severe disability, speech and language delay, behavioral problems (attention deficits and hyperactivity, impulsivity), congenital anomalies (ankyloglossia and undescended testicles), subtle facial features and vision impairment (refractive errors and strabismus) [9, 10]. SETBP1-HD was caused by the heterozygous deletion or loss of function (LoF) variant of *SETBP1* gene which resulted in nonsense-mediated mRNA decay (NMD) and haploinsufficiency [9]. SETBP1-HD had broad phenotypic spectrums and there were no specific genotype–phenotype correlations in the individuals of SETBP1-HD. There was no reasonable explanation for the variability of phenotype at present.

Many studies have shown that somatic *SETBP1* mutations were associated with hematologic malignancies [11–14]. The recurrent somatic activated mutations overlapped with the germline mutations which reported in congenital SGS. Studies have shown that somatic *SETBP1* mutations causing hematologic malignancies had greater driving effect to the degron than germline *SETBP1* mutations leading to SGS [15]. However, the potential carcinogenicity of the somatic *SETBP1* mutations remained unclear. Moreover, there were few reports on the relationship between somatic *SETBP1* mutation/expression and other tumors. In order to further unveil the underlying mechanisms of somatic *SETBP1* mutation/ expression leading to tumors, it was imperative to assess the expression patterns of the *SETBP1* gene in different tumors.

In this study, we reported a heterozygous novel de novo *SETBP1* mutation in a family and identified this mutation as the genetic cause of SETBP1-HD. Furthermore, we tried to reveal the relationship between *SETBP1* expression and SETBP1-HD. We gave a new view on the mental retardation of SETBP1-HD and performed a pan-cancer analysis of *SETBP1* gene in different tumors for the first time. We tried to have a deeper understanding of the mutation and expression pattern of *SETBP1* which might provide novel insights into related diseases.

## Materials and methods

### Patient description

The patient, 21-year-old, was born to unrelated healthy Chinese parents and was the second child of the family. One brother was phenotypically normal. The patient with mental retardation, expressive and receptive language skills impairment was 156 cm in height and 49 kg in weigh. She was pregnant and ultrasound examination showed the fetus with abnormal lateral fissure of the brain. She came to the Henan Provincial People’s Hospital for genetic counseling.

The written informed consent was obtained from her parents. This study was approved by the human and ethics committee of Henan Provincial People’s Hospital.

### DNA extraction, whole exome sequencing and data analysis

We extracted DNA from peripheral blood leukocytes by using a standard commercial kit (TIANGEN, Beijing, China) following the manufacturer’s instructions. The AIExomeV2 kit (iGeneTech Co., Beijing, China) was used for the Whole Exome Sequencing (WES). The whole exon region was enriched by liquid phase probe method and sequenced on Illumina Nova sequence platform (Illumina, Inc., California, USA) following the manufacturer’s standard operation instructions. In the study, 1% was used for the Minor allele frequency (MAF) threshold. The sequencing depth of the WES was 100× and the coverage of targeted exons reached 99%.

### Mutation confirmation

We performed Sanger sequencing to verify the mutations and identify the co-segregated of the mutations and the disease phenotype by ABI3500 Genetic Analyzer and Sequencing Analysis software (Applied Biosystems, Foster City, CA, USA). The primers were shown in Table 1.

**Table 1:** The sequence of the primers used

	Primer name	Primer sequence (5′-3′)
PCR	c.1724-1727del (F)	CCAACGTGCACAGATCACTC
	c. 1724-1727del (R)	CGTCGTCGCTTTCTTTTCTT
QPCR	SETBP1-E1-E2-F	CACAAAGCGGGCTAAGAAAC
	SETBP1-E1-E2-R	TAAGCCTGTGGCTGAAATCC
	SETBP1-E4-E5-F	CACCAGCCCCAGTGTTATCT
	SETBP1-E4-E5-R	GTTCACGTGGTCCAGGTTCT
RT-PCR	SETBP1-E3-1F	AAAGCCTTGGCTTCTGGAAT
	SETBP1-E3-1R	GTTTGCTTTCAATGGCTGCT

### Quantitative real-time PCR (QPCR)

The blood of the proband (D1), her mother (M1) and a normal unrelated individual control (X1) were collected for QPCR. Blood samples were collected in Blood RNA storage tubes (BIOTEKE, Beijing, China). The rapid extraction kit for total RNA of blood was used for RNA extraction (BIOTEKE, Beijing, China). cDNA synthesis was performed using HifairTM 1st Strand cDNA Synthesis SuperMix (YEASEN, Shanghai, China). CFX Connect Real-Time PCR Detection System (BioRad, CA, USA) and SYBR Green Realtime PCR Master Mix were used for QPCR (TOYOBO Co., Shanghai, China). Two pairs of primers were designed before and after the mutation. The primers were shown in Table 1.

### Western blotting (WB)

10 ml blood of the proband (D1), her mother (M1) and a normal unrelated individual control (X1) were collected for WB according to the method of our laboratory [16]. Anti-SETBP1 antibody used for WB was purchased from ABclonal Technology Co. (ABclonal, Wuhan, China) and was diluted according to the instruction. 293 T cells were used as positive control.

### The gene splicing array in vivo

Reverse transcription PCR (RT-PCR), cloning and sanger sequencing assay were used for gene splicing array in vivo of D1, M1 and X1. A pair of PCR primers across the mutation was designed on the third exon. The primers were shown in Table 1. The reaction conditions of RT-PCR were as follows: 95 °C, 5 min; 95 °C, 30 s; 57 °C, 30 s; and 72 °C, 30 s for a total of 30 cycles; 72 °C, 5 min. Agarose gel electrophoresis and sequencing was used for the evaluation of PCR products. For the products with overlapping peaks, we recycled the amplified products from the agarose gel. The amplified fragment was further connected to the pESI-T vector by using Hieff CloneTM Zero TOPO-TA Cloning Kit (Yeasen Biotech Co., Shanghai, China). Positive clones were sequenced. Rapid Plasmid Mini Kit was used for plasmid extraction and carried out in accordance with the instructions of the kit.

### mRNA sequencing (RNA-Seq)

The total RNA was collected by using a TRIzol reagent (Life technologies, MA, USA). RNA integrity was defined by Agilent 2100 Bioanalyzer (Agilent Technologies, CA, USA). cDNA Library construction was performed by using NEBNext® Ultra™ RNA Library Prep Kit for Illumina® Kit (NEB, MA, USA). After the library passed the inspection, different cDNA libraries were pooled according to the requirements of effective concentration and target offline data volume, and then sequencing was carried out on the Illumina HiSeq system (illumina, MA, USA).

### Cell culture and siRNAs for SETBP1

TH22, U87 and HMC3 cell lines were used for the follow-up assay. HMC3 cell line was purchased from GuangZhou Jennio Biotech Co.,Ltd (Jennio Co., Guangzhou, China). TH22 and U87 cell lines were a gift from Wuhan Botao Biotechnology Co., Ltd. TH22, U87 and HMC3 cell lines were grown in DMEM supplemented with 10% heat-inactivated FBS and 1% antibiotics maintained at 37 °C and 95% O_2_/5% CO_2_. Specific small interfering RNA (siRNA) was used for SETBP1 knockdown. We designed three siRNAs. SETBP1 siRNA #1 sequence:5′-CCCUAUGGAAUGCCUUACATT-3′; SETBP1 siRNA #2 sequence: 5′-CGGUGCCAUAUAUCCAGUATT-3′; SETBP1 siRNA #3 sequence: 5′-GGAGCUGCCAACUGGUCAATT-3′. The final transfected concentration of the siRNAs was 100 mM/L. The Lipofectamine 2000 and Opti-MEM® (Thermo Fisher Scientific, MA, USA) were used for transfection.

### Transwell matrigel and wound healing assays

Cell migration was assessed by transwell and wound healing assays. HMC3 cells were transfected with SETBP1 siRNA oligonucleotides and inoculated in a 6-well plate at a density of 2 × 10^5^ cells/well, and cultivated overnight in an incubator at 37 °C and 5% CO_2_ before cell matrigel assays.

HMC3 cells were plated onto the upper part of a transwell (BD, NJ, USA) at a density of 1 × 10^5^ cells/well and maintained for 24 h at 37 °C in the incubator. The bottom of the transwells were supplied with 800 μL 1640 medium containing 10% FBS. After 24 h of treatment, non-migrated cells were gently removed with a cotton swab. The migrated cells were treated as follows: 70% ethanol solution for 1 h and staining with 0.5% crystal violet for 20 min at room temperature. The migrated cells then were photographed by using the inverted microscope (MSHOT, Guangzhou, China) and counted for 4 randomly selected fields in each well. Cell migration was expressed using the number of the cells.

The above HMC3 cells (transfected with siRNAs and control) were wounded with a sterile scratcher. The cell migration was determined by measuring the changes in the area of the wounds at 0 and 24 h using the inverted microscope (MSHOT, Guangzhou, China).

### Cell apoptosis arrays by flow cytometry

The fluorescein isothiocyanate (FITC)-annexin V apoptosis detection kit (KeyGEN BioTECH, Nanjing, China) was used for cell apoptosis according to the manufacturer’s protocols. HMC3 cells were collected after transfection for 24 h (siRNA #3) and resuspended by annexin binding buffer. And then the cells were added with annexin V and propidium iodide (PI) and incubated at room temperature for 15 min in dark. After 1 h, they were analyzed by a flow cytometer (Beckman CytoFLEX, CA, USA).

### Mice

C57BL/6 J mice (8–10 weeks old) were purchased from Beijing Vital River Laboratory Animal Technology Co., Ltd. Mice were bred and maintained in specific pathogen–free conditions. C57BL/6 J × C57BL/6 J mating combinations were established. The brains of the mice were taken from embryonic day 11 (E11), E15, postnatal day 1 (P1), P3, P5 and P7. The *SETBP1* expression profiles were examined by using QPCR. The sequences of the primers were as follows: GAPDH-F, 5′-ATGGGTGTGAACCACGAGA-3′, GAPDH-R, 5′-CAGGGATGATGTTCTGGGCA-3′; SETBP1-F, 5′-CAAACCTCCGGCTATGCTTC-3′, SETBP1-R, 5′-AACTCTCTGCTGATTGGGCT-3′.

### *SETBP1* expression and mutation profile and its correlation with genomic heterogeneity

The consensus dataset consisting of normalized expression (nTPM) levels for 55 tissue types was created by HPA and GTEx transcriptomics datasets. The tumor immune estimation resource, version 2 (TIMER2.0) online tools were used to analyze the expression of *SETBP1* in different tumor tissues. The gene expression profiling interactive analysis version 2 (GEPIA2) online program was used to analyze the expression of *SETBP1* gene in ACC, DLBC, HNSC, LAML, LGG, OV, SARC, SKCM, TGCT and UCS (with the cut-off criteria of |Log_2_FC|> 1 and adjusted *P* value ≤ 0.01) and normal tissues were matched from TCGA and GTEx database. GEPIA2 was used to analyze the expression of *SETBP1* in different tumor stages.cBioPortal software was used to map the location of the mutations observed in *SETBP1* gene in different types of tumors. The pan cancer dataset was obtained from UCSC database: TCGA pan cancer (PanCAN, n = 10,535, g = 60,499). Further, we extracted the expression data of *SETBP1* (ENSG00000152217) gene in samples, and screened the samples from primary blood derived cancer—peripheral blood and primary tumor from the above pan cancer dataset. The CNV data (processed by GISTIC software [17]) and the SNP data (processed by MuTect2 software [18]) of all TCGA samples in level 4 were obtained from GDC database. We integrated the gene expression data and CNV data/SNP data of the samples obtained above, filtered the samples with expression level of 0, transformed each expression value with log2 (x + 0.001), eliminated the cancers with less than 3 samples in a single cancer species. Finally, we obtained the CNV data of 23 cancers and SNP data of 22 cancers. The expression difference of *SETBP1* in different clinical stage samples in each tumor was calculated by R package (version 3.6.4). Pairwise significance analysis was performed using unpaired Wilcoxon Rank Sum and Signed Rank Tests. The significance analysis between groups was performed using kruskal test.

Tumor Mutation Burden (TMB) was calculated using the tmb function of the R package maftools (version 2.8.05). Microsatellite Instability (MSI) score of each tumor was obtained from the previous research [19]. We integrated the gene expression data and TMB data/MSI data of the samples obtained above, filtered the samples with expression level of 0, transformed each expression value with log2 (x + 0.001), eliminated the cancers with less than 3 samples in a single cancer species, the TMB data of 37 cancers and MSI data of 37 cancers were obtained finally.

### Immune cell infiltration and survival analysis

Six algorithms (TIMER, CIBERSORT, quanTIseq, xCell, MCP-counter and EPIC) of TIMER2 software were used to evaluate the correlation between *SETBP1* expression level and immune cell infiltration level (CD8+T-cells and cancer-associated fibroblasts). The overall survival (OS) and disease free survival (RFS) analysis of *SETBP1* in different tumors were performed by GEPIA. Samples were classified into high expression and low expression groups based on the 50% (median) cutoff. The log-rank test was used to assess the significant difference of survival curves of two cohorts.

### Functional enrichment analysis

Similar genes of *SETBP1* in normal tissues in the GTEX were detected by GEPIA2 software. The gene annotation of the latest Kyoto encyclopedia of genes and genomes (KEGG) pathway was obtained from the KEGG rest API (https://www.kegg.jp/kegg/rest/keggapi.html). The c5.go.bp.v7.4.symbols.gmt, c5.go.mf.v7.4.symbols.gmt and c5.go.cc.v7.4.symbols.gmt subsets were obtained from the Molecular Signatures Database (DOI:10.1093/bioinformatics/btr260). We performed KEGG and gene ontology (GO) enrichment analysis by using the R package clusterProfiler (version 3.14.3).

## Results

### Clinical report

**Fig. 1:**
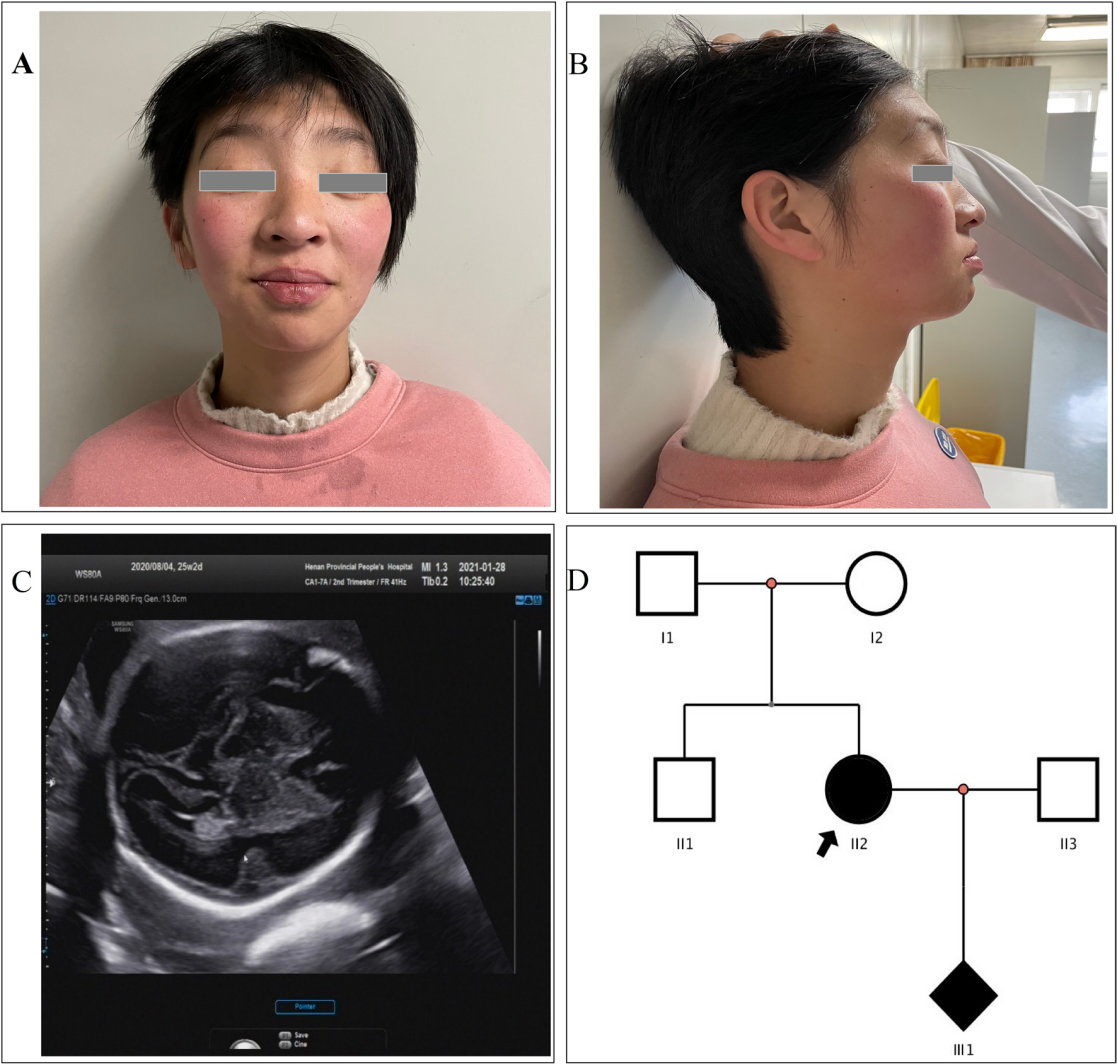
The clinical data and family information. **A** The front photo of the patient’s special facial features. **B** The side photo of the patient’s special facial features. **C** The fetal ultrasound showed abnormal lateral fissure of the brain. **D** The family pedigree. Filled symbols refer to subjects with *SETBP1* mutation (c.1724_1727 del, p.D575Vfs*4)

The patient was referred to our genetic counseling clinic for mental retardation, expressive and receptive language skills impairment and specific facial features (Fig. 1A, B). The patient was 156 cm in height and 49 kg in weigh. She was pregnant and the fetus was found abnormal lateral fissure in the brain (Fig. 1C). She was the second child of healthy unrelated Chinese parents with one healthy brother (Fig. 1D). She was born after an uneventful term pregnancy. After birth, the patient can erect her head at 3 months, sit alone at the age of 6 months, and walk independently at 1 year and 5 months. Her language development was extremely backward so that she could only say two simple words: father and mother as of now. She also had the stereotyped movement of touching her lower lip. From the physical examination results, we noted her peculiar facial features: long face, high forehead, small palpebral fissures with ptosis, bilateral epicanthal folds, broad nasal tip, thin upper lip, fleshy lower lip and blush on both cheeks. No obvious abnormalities were found in routine blood test, routine coagulation test, cardiac color ultrasound and digestive system ultrasound.

### Identification of a de novo heterozygous mutation of the *SETBP1* gene

**Fig. 2:**
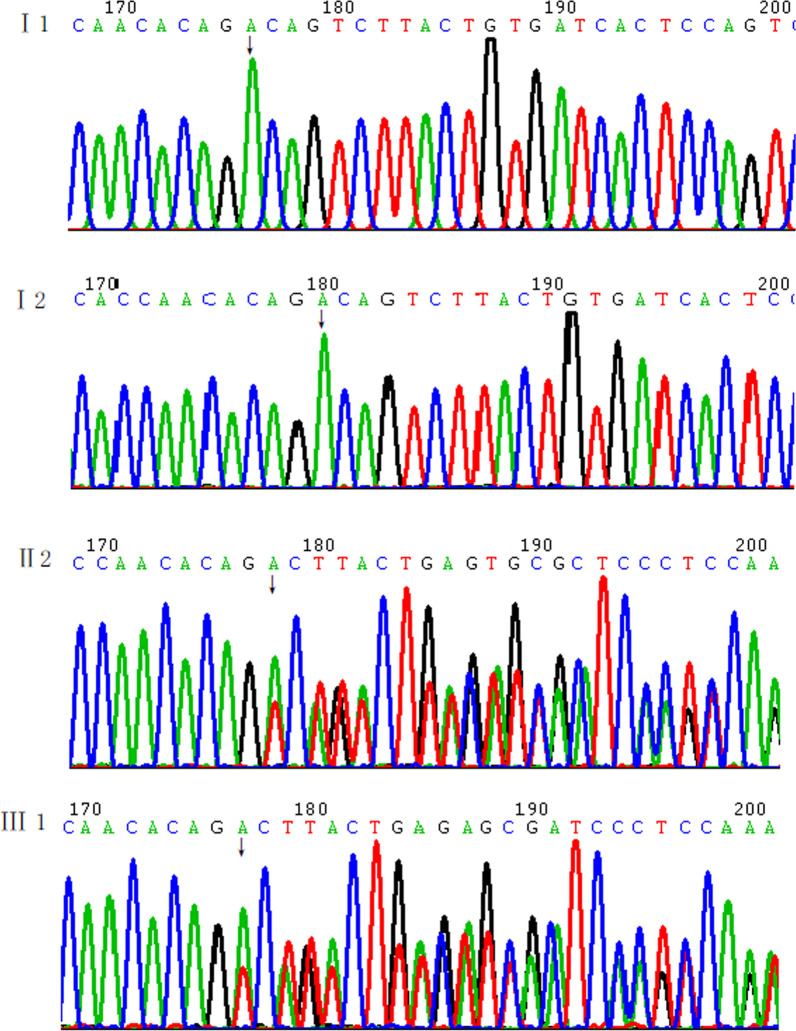
The Sanger sequencing results of the patient, her fetus and her parents of the mutation (*SETBP1* c.1724_1727del)

We detected the patient, her parents and the fetus by using WES. By excluding synonymous variants, a novel heterozygous mutation of the *SETBP1* gene (c.1724_1727del, p.D575Vfs*4) which located in the third exon was found in the patient and the fetus and the mutation was predicted to result in a truncated protein. Sanger sequencing confirmed the mutation. The results determined the mutation was de novo in the patient (Fig. 2).

### The expression and splicing of *SETBP1* in vivo

**Fig. 3:**
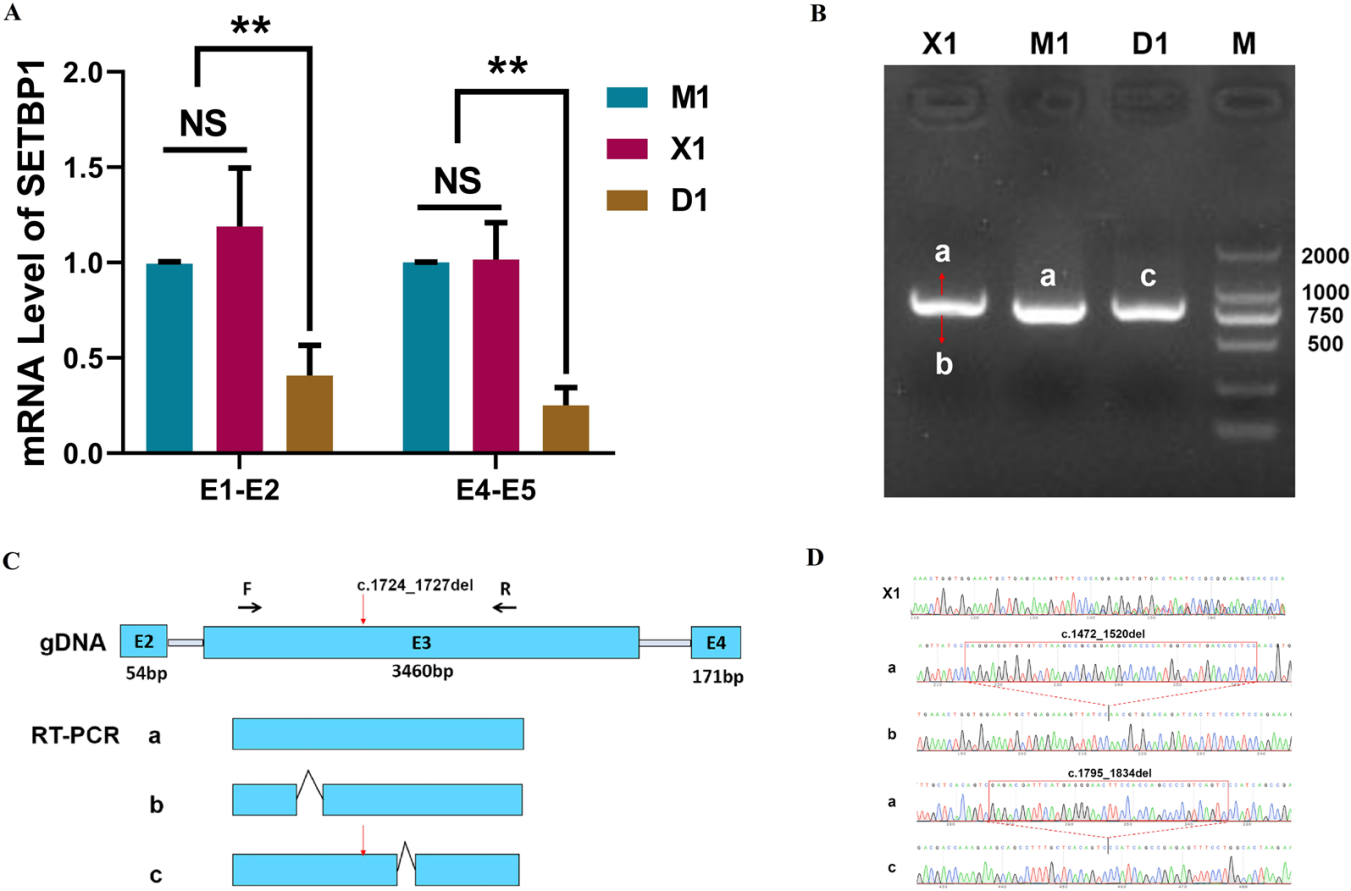
The expression and splicing of *SETBP1* in vivo. **A** The mRNA expression level of *SETBP1* in D1, M1 and X1. **B** The results of agarose gel electrophoresis of RT-PCR. **C** The schematic diagram of the splicing of SETBP1 in vivo in D1, M1 and X1. **D** The cloning and sequencing results of X1 and the sequencing results of D1 and M1. Except for the mutation (*SETBP1* c.1724_1727del), a 40 bp deletion in D1 (band c) was found (c.1795_1834del). A 49 bp deletion (c.1472_1520del) was found in X1 (band b)

The blood of the proband (D1), her mother (M1) and a normal unrelated individual control (X1) were collected for the array. Two pairs of primers for QPCR were designed before and after the mutation. We detected a decrease of the *SETBP1* mRNA in D1 comparing to M1 and X1 (Fig. 3A). We failed to detect SETBP1 protein in the blood of the three samples. The loading volume of the WB was 40 μg, and the reference bands (Actin) in three samples were visible. At the same loading amount, we could see the target protein fragment in the control 293 T cells. This indicated that the expression of the *SETBP1* gene in blood might be relatively low.

We designed a pair of PCR primers across the mutation on the third exon for the splicing of *SETBP1* array in vivo. The results of agarose gel electrophoresis of PCR products were shown in Fig. 3B. The sequencing results of PCR products of three samples showed that X1 was a nested sequencing peak, M1 was a normal band and D1 only detected an abnormal band (Fig. 3C, D). There was a 40 bp deletion of D1 (band c) at 67 bp from the mutation (c.1795_1834del), resulting in the change of protein coding sequence to p.Asp575ValfsTer4. We connected the X1 PCR product to the vector, cloned and sequenced. The sequencing results showed that there were two splicing modes in X1, named a-band and b-band respectively. A-band was a normal splicing band, and b-band had a 49 bp deletion (c.1472_1520del), resulting in the change of protein coding sequence as p.Pro491GlnfsTer48. No mutations in exon 3 of *SETBP1* were found in X1. We speculated that the b-band may be a new splice or transient. No normal band was detected in the patient D1, which may be due to the low expression of normal band in the patient.

### The mRNA profile in the patient and controls were different

**Fig. 4:**
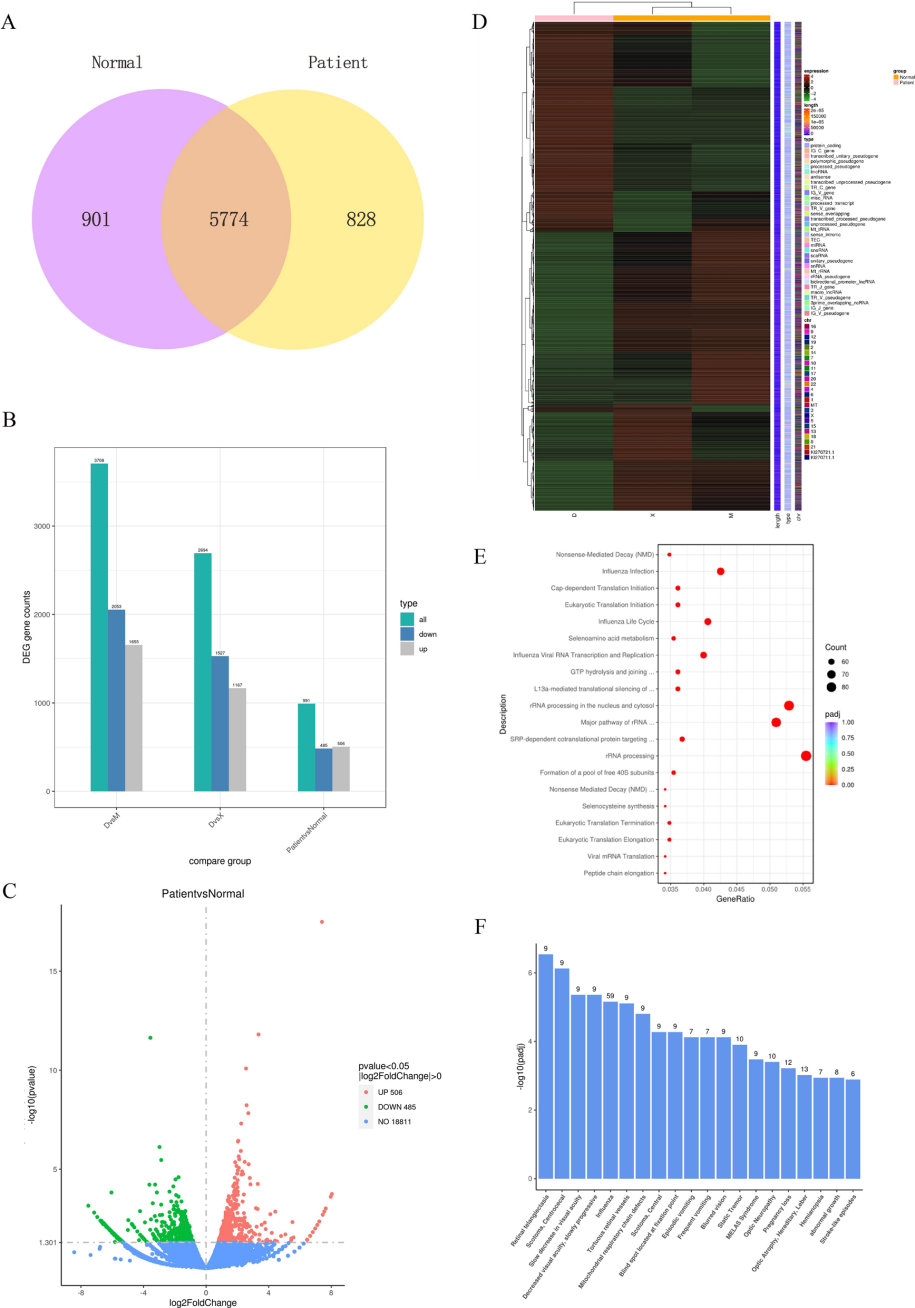
Overview of mRNA profile in D1, M1 and X1. **A** Venn diagram showed the number of genes uniquely expressed and CO expressed in each group. **B** Columnar statistical chart showed the number of differentially expressed genes between the patient and controls. **C** Volcano Plot showed differentially expressed genes between the patient and controls (*P* value < 0.05, |log2FoldChange|> 0). **D** The Heatmap conducted according to the RNA-seq data. **E** The scatter plots showed the top 20 significantly different terms of reactome pathway enrichment analysis. **F** The Histogram showed the top 20 significantly different terms of DisGeNET analysis

Studies have shown that SETBP1 could bind to gDNA in AT-rich promoter regions and then affected the activation of gene expression [20]. From the results of RNA-seq in the D1, M1 and X1, we found that there was a significant difference between the patient (D1) and controls (M1 and X1) in the genome-wide expression profile. We performed quantitative analysis of gene expression. We drew the Venn diagram to show the number of genes which uniquely expressed and CO expressed in each group/sample. There were 828 and 901 genes uniquely expressed in the patient and controls respectively (Fig. 4A). Compared with the controls, we screened 991 mRNAs with significant differences in expression levels, including 506 up-regulated mRNAs and 485 down-regulated mRNAs (*P* value < 0.05, |log2FoldChange|> 0). The Columnar statistical chart and Volcano Plot were conducted and visually showed the differences between the patient and controls (Fig. 4B, C). After merging the differential genes of all comparison groups as the differential gene set, we carried out cluster analysis and drew a Heatmap (Fig. 4D) which showed the chromosome, gene length and biological type of each differential gene.

We used cluster profiler software for GO analysis and KEGG analysis of differential gene sets. GO and KEGG scatter plots showed the top 30 and the top 20 significantly different terms respectively (Additional file 1: Fig. S1A, B). We also performed reactome pathway enrichment analysis and DisGeNET analysis. The scatter plots showed the top 20 significantly different terms of reactome pathway enrichment analysis (Fig. 4E). The Histogram showed the top 20 significantly different terms of DisGeNET analysis (Fig. 4F).

### SiSETBP1 inhibited the invasion and proliferation of HMC3 and increased the apoptosis of HMC3

**Fig. 5:**
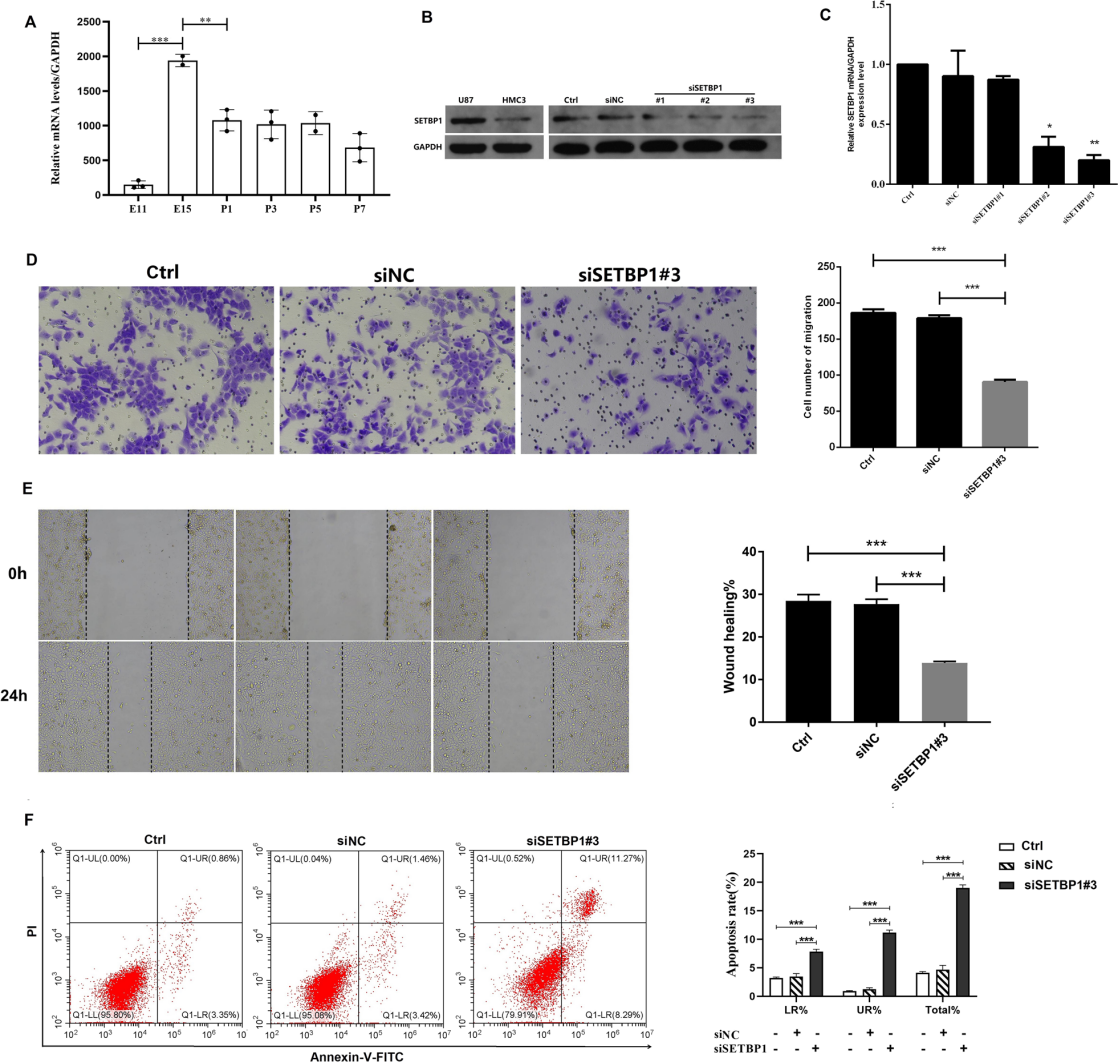
SiSETBP1 was able to inhibit the invasion and proliferation of HMC3 and increase the apoptosis of HMC3. And in the mice brains the expression peak of *SETBP1* occurred in about E15. **A** qRT-PCR analysis of *SETBP1* in the mice brains taken from embryonic day 11 (E11), E15, postnatal day 1 (P1), P3, P5 and P7. Two adjacent groups were compared. **B** WB was used to detect the *SETBP1* expression of HMC3 cell line with *SETBP1* knockdown. GAPDH was used as an internal reference. U87 was used as positive control. **C** qRT-PCR analysis of *SETBP1* of HMC3 cells with *SETBP1* knockdown. **D** Representative photomicrographs of the transwell migration. **E** Representative photomicrographs of the wound healing assay were taken over time at 0 and 24 h. Black dashed lines delineate the wound area. **F** HMC2 Cell apoptosis were analyzed by flow cytometry assay 24 h after treatment of siSETBP1#3. Ctrl = control, siNC = small interfering normal control. *P < 0.05, **P < 0.01, ***P < 0.001

To understand the expression of *SETBP1* in the mice brains, the expression of *SETBP1* in the mice brains of embryonic day 11 (E11), E15, postnatal day 1 (P1), P3, P5 and P7 were detected. Two adjacent groups were compared. We found the expression peak of *SETBP1* occurred in about E15 (Fig. 5A). In order to illustrate the role of *SETBP1* in microglia, we selected the HMC3 cells transfected with siSETBP1 for matrigel invasion assays, wound healing assays and cell apoptosis arrays. The third siSETBP1 oligonucleotide (#3) was the best one for *SETBP1* knockdown (Fig. 5B, C). U87 was used as positive control. The migration invasion assays showed that interference of the *SETBP1* expression significantly inhibited the invasion of HMC3 cells (Fig. 5D). The wound healing assays showed that interference of the *SETBP1* expression significantly inhibited the proliferation of HMC3 cells (Fig. 5E). Flow cytometry expreriments confirmed that the apoptosis rate of HMC3 increased when it was treated with siSETBP1#3 (Fig. 5F). These findings suggested that the siSETBP1 inhibited the invasion and proliferation of HMC3 and increased the apoptosis of HMC3.

### *SETBP1 *expression, mutation profile and correlation analysis of *SETBP1* expression and genomic heterogeneity

**Fig. 6:**
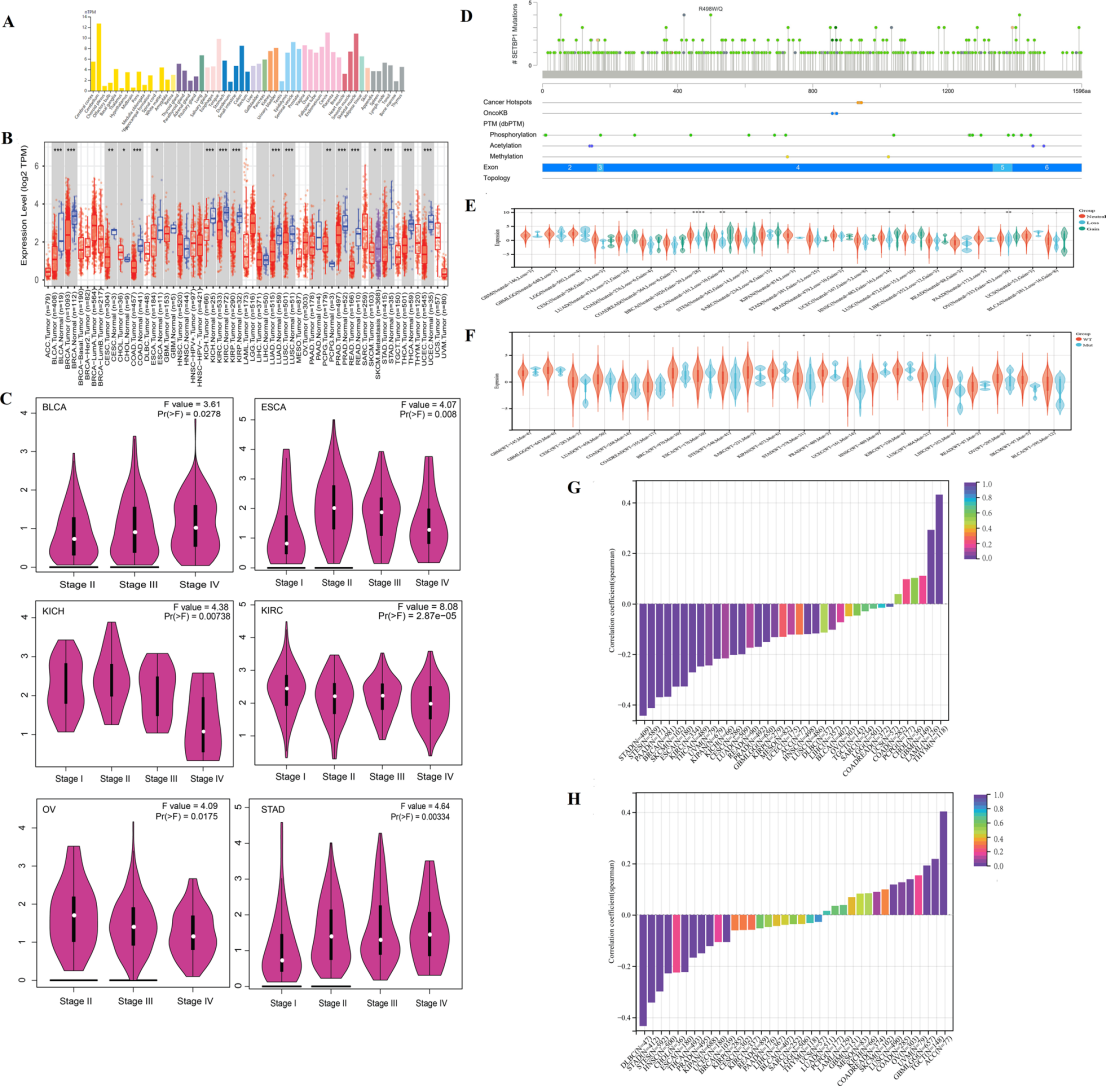
*SETBP1* expression, mutation profile and correlation with genomic heterogeneity. **A** The expression profile of *SETBP1* gene in 55 normal tissues. **B** The expression of *SETBP1* gene in tumor tissues and the comparison with normal tissue. **C** The expressions of *SETBP1* gene in different tumor stages and showed 6 tumors with significantly different in different tumor stages (*P* < 0.05). **D** *SETBP1* mutation profile in tumors. **E** CNV profile in 23 tumors and the comparison with normal tissue.** F** CNV profile in 22 tumors and the comparison with normal tissue. **G** The correlation analysis of *SETBP1* expression and TMB in 37 tumors. **H** The correlation analysis of *SETBP1* expression and MSI in 37 tumors

The expression profile of *SETBP1* gene in 55 normal tissues was showed in Fig. 6A. We could see that *SETBP1* had the highest expression level in the cerebellum, followed by skeletal muscle and tissues of the reproductive system. The full name and abbreviations of tumors were showed in Additional file 4: Table S1. The expression of *SETBP1* gene in 33 tumor tissues was showed in Fig. 6B and Additional file 2: Fig. S2A. The *SETBP1* expressions of tumor tissues were compared to the normal tissues of the same origin, except of MESO, THYM and UVM in which the *SETBP1* expression data was missing. We found that the expressions of *SETBP1* gene in BLCA, BRCA, CESC, CHOL, COAD, ESCA, KICH, KIRC, KIRP, LUAD, LUSC, PCPG, PRAD, READ, SKCM, STAD, THCA, UCEC, OV and UCS were significantly different from that in normal tissues (*P* < 0.05). The expressions of *SETBP1* gene in different tumor stages of 23 tumors were showed in Fig. 6C and Additional file 2: Fig. S2B. The expressions of *SETBP1* gene in different tumor stages of BLCA, ESCA, KICH, KIRC, OV and STAD were also significantly different (*P* < 0.05).

The *SETBP1* mutation profile in tumors was showed in Fig. 6D. Missense mutations, truncating mutations and splice mutations were found. CNV analysis in 23 tumors was showed in Fig. 6E. We observed significant differences in 7 tumors: LUAD (*P* = 0.02), BRCA (*P* = 5.3e−6), ESCA (*P* = 8.7e−3), STES (*P* = 0.02), HNSC (*P* = 0.04), LUSC (*P* = 0.02), OV (*P* = 3.0e−3). SNP analysis in 22 tumors was showed in Fig. 6F. We observed significant differences in 4 tumors: STAD (*P* = 0.03), HNSC (*P* = 1.3e−3), LUSC (*P* = 7.7e−3) and SKCM (*P* = 5.8e−3). These data indicated that there were different mutation types of *SETBP1* in different tumors.

We analyzed the correlation between *SETBP1* expression and TMB (Spearman correlation) in 37 tumors, and observed significant correlation in 19 tumors. The results were showed in Fig. 6G. Two tumors were in significant positive correlations: LAML (R = 0.2920, *P* = 0.0011) and THYM (R = 0.4317, *P* = 0.0000). Seventeen tumors were in significant negative correlation: GBMLGG (R = − 0.1511, *P* = 0.0001), CESC (R = − 0.2024, *P* = 0.0006), LUAD (R = − 0.1996, *P* = 0.0000), BRCA (R = − 0.3683, *P* = 0.0000), ESCA (R = -0.3275, *P* = 0.0000), STES (R = − 0.4131, *P* = 0.000), KIRP (R = − 0.1319, *P* = 0.0276), KIPAN (R = − 0.2177, *P* = 0.0000), STAD (R = − 0.4435, *P* = 0.0000), PRAD (R = − 0.1704, *P* = 0.0001), HNSC (R = − 0.1200, *P* = 0.0073), KIRC (R = − 0.2716, *P* = 0.0000), LUSC (R = − 0.1175, *P* = 0.0096), THCA (R = − 0.2483, *P* = 0.0000), PAAD (R = − 0.3702, *P* = 0.0000), SKCM (R = − 0.3281, *P* = 0.0008) and UVM (R = − 0.2442, *P* = 0.0301). The correlation analysis of *SETBP1* expression and MSI (Spearman correlation) in 37 tumors was showed in Fig. 6H. We observed significant correlations in 15 tumors. Six tumors were in significant positive correlations: GBMLGG (R = 0.1925, P = 0.0000), COAD (R = 0.1269, *P* = 0.0322), LUSC(R = 0.1181, *P* = 0.0089), OV (R = 0.1387, *P* = 0.0157), TGCT (R = 0.2180, *P* = 0.0078) and ACC (R = 0.4030, *P* = 0.0003). Nine tumors were in significant negative correlation: BRCA (R = − 0.1059, *P* = 0.0006), ESCA (R = − 0.2231, *P* = 0.0026), STES (R = − 0.2985, *P* = 0.0000), KIPAN (R = − 0.1218, *P* = 0.0014), STAD (R = − 0.3419, *P* = 0.0000), PRAD (R = − 0.1499, *P* = 0.0008), HNSC (R = − 0.2280, *P* = 0.0000), THCA (R = − 0.1666, *P* = 0.0002) and DLBC (R = − 0.4335, *P* = 0.0023). These data suggested that the expression level of *SETBP1* was inconsistent with genomic heterozygosity (MSI and TMB) in different tumors.

### *SETBP1* expression correlated with immune cell infiltration and survival rate

**Fig. 7:**
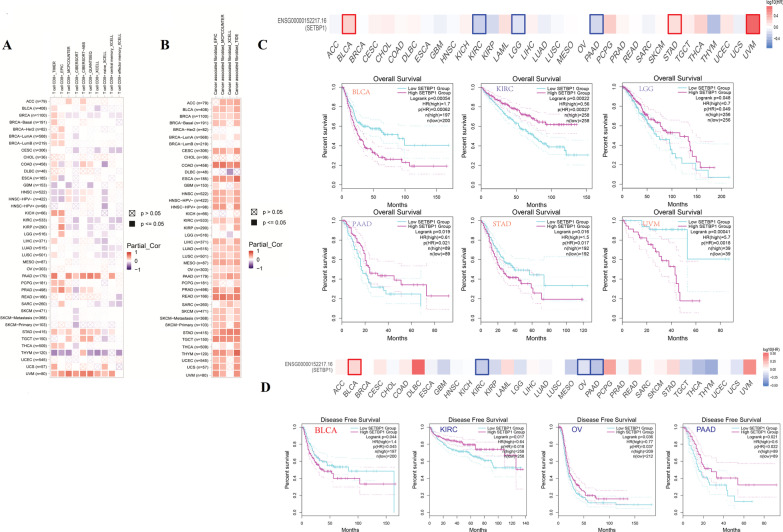
Immune cell infiltration and survival analysis. **A** and **B** The correlation analysis of *SETBP1* expression and the immune cell infiltration (CD8^+^ T-cells and cancer-associated fibroblasts). **C** and **D** The correlation analysis between *SETBP1* expression and the survival (Overall Survival and Disease Free Survival)

The correlation analysis between the expression level of *SETBP1* and the level of immune cell infiltration (CD8^+^ T-cells and cancer-associated fibroblasts) were showed in Fig. 7A, B. We observed 2 tumors were in significant positive correlations (PAAD and UVM) and 1 tumor was in significant negative correlations (THYM). In most tumors, we observed no correlation of T cell infiltration with the expression level of *SETBP1* gene. On the contrary, we observed positive correlations between the expression level of *SETBP1* and the level of cancer-associated fibroblasts in most tumors.

The correlation analysis between the expression level of *SETBP1* and the survival (OS and RFS) were showed in Fig. 7C, D. The high expression level of *SETBP1* reduced the OS in patients of BLCA, STAD and UVM. The low expression level of *SETBP1* reduced the OS in patients of KIRC, LGG and PAAD. At the same time, we observed that the high expression level of *SETBP1* reduced the disease free survival in patients of BLCA and low expression level of *SETBP1* reduced the disease free survival in patients of KIRC, OV and PAAD. These data indicated the survival rate of some tumors were correlated with *SETBP1* expression level.

### *SETBP1*-related genes and related pathways involved in tumor genesis

**Fig. 8:**
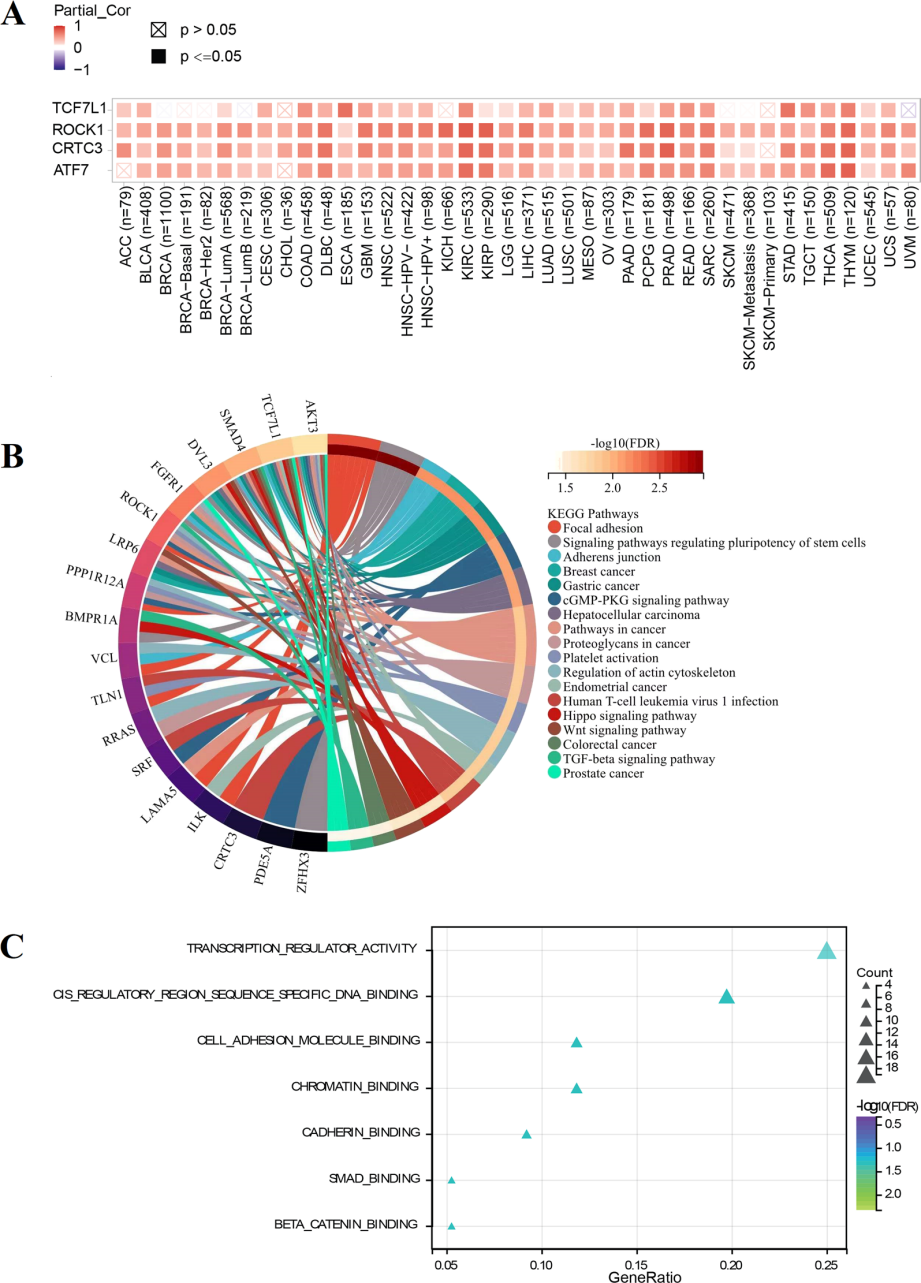
Functional enrichment analysis. **A** The expression patterns of the first four similar genes compared with that in different tumors. **B** The top 18 enriched KEGG pathways. **C** The 7 enriched GO terms with significant difference

We screened gene sets with similar expression pattern of *SETBP1* used normal tissues in GTEX (Top 100). The expression patterns of the top four similar genes were showed in Fig. 8A. We could see that the expression pattern of these genes differed between normal and tumors. The results of KEGG and GO analysis were showed in Fig. 8B, C and Additional file 3: Fig. S3. Among the top 18 enriched KEGG pathways, 8 KEGG pathways were related to the occurrence of cancers, such as breast cancer, gastric cancer, hepatocellular carcinoma, pathways in cancer, proteoglycans in cancer, endometrial cancer, colorectal cancer and prostate cancer. GO analysis revealed that *SETBP1*-related genes were associated with the pathway of transcription regulator activity, cis regulatory region sequence specific DNA binding, cell adhesion molecule binding, chromatin binging and so on. *SETBP1*-related genes associated with the Cellular Component (CC) and Biological Process (BP) were showed in Additional file 3: Fig. S3.

## Discussion

In the past decade, *SETBP1* has attracted a lot of interest on that the same gene with different type or level (germline or somatic) of mutations that could provoke different pathologic consequences. *SETBP1* was discovered in 2001 and the *SETBP1* mutations were found for the first time in a germline congenital disease called SGS [1, 2]. Most of the germline *SETBP1* mutations in SGS occurred in a mutational hotspot region (codons 858–971). This region was part of a degron motif which contained a consensus-binding region for SCF-β- TrCP E3 ubiquitin ligase. The mutated *SETBP1* occurred to be a functional loss of binding the E3 ligase subunit. That may trigger an increased SETBP1 protein stability and its accumulation which may directly inhibit PP2A through SETBP1-SET-PP2A axis [4]. Recently, authors suggested that *SETBP1* can bind to gDNA in AT-rich promoter regions, provoking transcription of a set of development genes through recruitment of a HCF1/KMT2A/PHF8 epigenetic complex [20]. All in all, these studies unveiled the mechanism operating in SGS and myeloid malignancies and supported the contention gain-of-function or a dominant-negative effect [2, 5, 21]. SGS featured multisystemic involvement with severe fatal developmental syndrome and the reported SGS patients had highly overlapping clinical phenotypes and recognizable. This study will not go into too much detail.

Unlike gain-of-function or a dominant-negative effect, haploinsufficiency or loss-of-function mutations of *SETBP1* could cause SETBP1-HD and produce a mild to moderate phenotype. SETBP1-HD had broad phenotypic spectrums and there were no specific genotype–phenotype correlations. The variability in SETBP1-HD phenotype and relatively prominent nervous system phenotype was no reasonable explanation at present. In this study, we identified a de novo heterozygous mutation of the *SETBP1* (c.1724_1727del, p.D575Vfs*4) and confirmed the mutation could lead to decreased expression of *SETBP1.* From the results of RNA-seq conducted in the patient and controls, we found that there was a significant difference between the patient and controls in the genome-wide expression profile. The result of reactome pathway enrichment analysis showed that there were significant differences in the pathway of NMD. This was consistent with the previous studies and our experimental results of QPCR [9, 21].

We were particularly concerned about the mild to moderate neurological symptoms that were almost always present in these SETBP1-HD patients in the present study. The neuronal cell line HT22 and human microglial cell line HMC3 were selected to study the effects of *SETBP1* haploinsufficiency. As we all know, the neuronal cell line HT22 was an excellent model for studying Parkinson's disease [22]. However, we did not detect *SETBP1* expression in HT22 cell line. Whether microglia playing a key role in the nervous system phenotypes of *SETBP1* related diseases needed further in vivo experiments. As the brain-resident macrophages of the central nervous system (CNS), microglia played important roles in innate immunity, neuroinflammatory pathologies and regulated brain development [23]. Microglia was derived from erythroid precursor cells in the yolk sac tissue. They migrated, colonized, differentiated, and matured in the brain, and eventually reached a steady state in the CNS environment. Compared with adult mature microglia, embryonic microglia exhibited great heterogeneity and were involved in the regulation of various physiological activities. The microglial cells could affect CNS by modulating neurons synapse formation and neuronal survival [24, 25]. We endeavored to shed light on the mechanisms by which *SETBP1* expression in microglial cells exerted its role in CNS development of SETBP1-HD. The effects of knock-down expression were studied by conducting a series of cell experiments using HMC3 cell line and the brain tissue of the mice. The novel findings of this study included: (1) *SETBP1* knock-down in HMC3 increased the apoptosis of HMC3; (2) *SETBP1* knock-down in HMC3 resulted to a significant reduction in the migration and proliferation of HMC3; (3) The expression level of *SETBP1* in the brains of mice was detected. We found the expression peak of *SETBP1* occurred in about E15. These results indicated that *SETBP1* may play a key regulatory role in the proliferation and migration of microglia cells. In addition, the findings supported the *SETBP1* insufficiency may involve in the brain development as early as the embryonic period. In the clinical case we studied, the fetus in the second trimester of pregnancy with *SETBP1* mutation was found with abnormal lateral fissure of the brain. The findings laid the foundation for understanding the processes implicated in the brain development of SETBP1-HD.

Somatic mutations of *SETBP1* were associated with myeloid malignancies due to its gain-of-function or a dominant-negative effect. The changes observed in the patients of *SETBP1* mutations can be considered functionally equivalent to *SETBP1* overexpression [4, 26]. In the past, scholars focused on the relationship between *SETBP1* mutations and tumors, especially myeloid malignancies and *SETBP1* mutations was served as a biomarker for the diagnosis and poor prognosis of myeloid malignancies and the overlap syndrome [27]. There were few reports on the relationship between somatic *SETBP1* expression and other tumors. It was imperative to assess expression patterns of *SETBP1* and correlation with tumors. A pan-cancer analysis of *SETBP1* was conducted based on previous reported data sets for the first time. We found that the expressions of *SETBP1* in BLCA, ESCA, KICH, KIRC, OV and STAD were significantly different from those in normal tissues and in different tumor stages. Among the six tumors, ESCA was found in significant negative correlation from the results of correlation analysis of *SETBP1* expression and TMB/MSI. The correlation analysis between the expression level of *SETBP1* gene and OS showed that the high expression level of *SETBP1* reduced the OS in patients of BLCA, STAD and UVM. At the same time, we observed that the high expression level of *SETBP1* reduced the RFS in BLCA patients. In a large study of 727 patients with various myeloid malignancies, *SETBP1* mutations were found in 52 cases (7.2%) [28]. Somatic mutations of *SETBP1* resulted in gain-of-function were associated with poor prognosis and myeloid leukemic transformation in patients with myelodysplastic syndromes (MDS) and chronic myelomonocytic leukemia (CMML) [4, 29]. *SETBP1* mutations may be a biomarker for the diagnosis and OS for the above syndrome and myeloproliferative neoplasm (MPN) overlap syndrome [27]. In any case of malignant tumors the OS was poor. Physicians should be aware of the high risk of malignancies in these patients with *SETBP1* mutations or *SETBP1* overexpression. Similarly, our findings suggested that *SETBP1* overexpression should be given special attention in BLCA and STAD. However, tumors had different subtypes, we only focused on the tumor reported in the public database which some subtypes may not be included in. Caution should be taken into the correlation between the expression of *SETBP1* and tumors of different subtypes.

In conclusion, both *SETBP1* germline and somatic mutations could lead to allele dose changes at molecular level which highlighted that it was a dose sensitive gene. *SETBP1* overexpression was associated with SGS and myeloid malignancies. Reduced *SETBP1* expression was associated with SETBP1-HD. Association analyses between *SETBP1* expression levels with clinical survival analysis, genomic heterozygosity and immune infiltration in different cancers revealed its complex role in the pathogenesis of cancers. Whether the *SETBP1* expression or mutations could be used as a biomarker for the diagnosis and poor prognosis of tumors was worth further studied.

## Supplementary Information

**Additional file 1. Figure S1**: GO and KEGG scatter plots. **A** The top 30 GO terms with significant difference. **B** The top 20 KEGG terms with significant difference.

**Additional file 2. Figure S2**: The expression of SETBP1 gene in tumor tissues and different tumor stages. **A** The expression of SETBP1 gene in tumor tissues. **B** The expressions of SETBP1 gene in different tumor stages.

**Additional file 3. Figure S3**: The enriched GO terms with significant difference. **A** SETBP1-related genes associated with the cellular component with significant difference. **B** SETBP1-related genes associated with the biological process with significant difference.

**Additional file 4. Table S1**: The abbreviation and full name of the tumors.

## Data Availability

The datasets generated during and/or analysed during the current study are available from the corresponding author on reasonable request.
