# Supplementary figures and images for: Identification of a novel de novo mutation of SETBP1 and new findings of SETBP1 in tumorgenesis
Source: Orphanet J Rare Dis. 2023 May 7;18:107. doi: 10.1186/s13023-023-02705-6 (PMC10165755; doi:10.1186/s13023-023-02705-6)

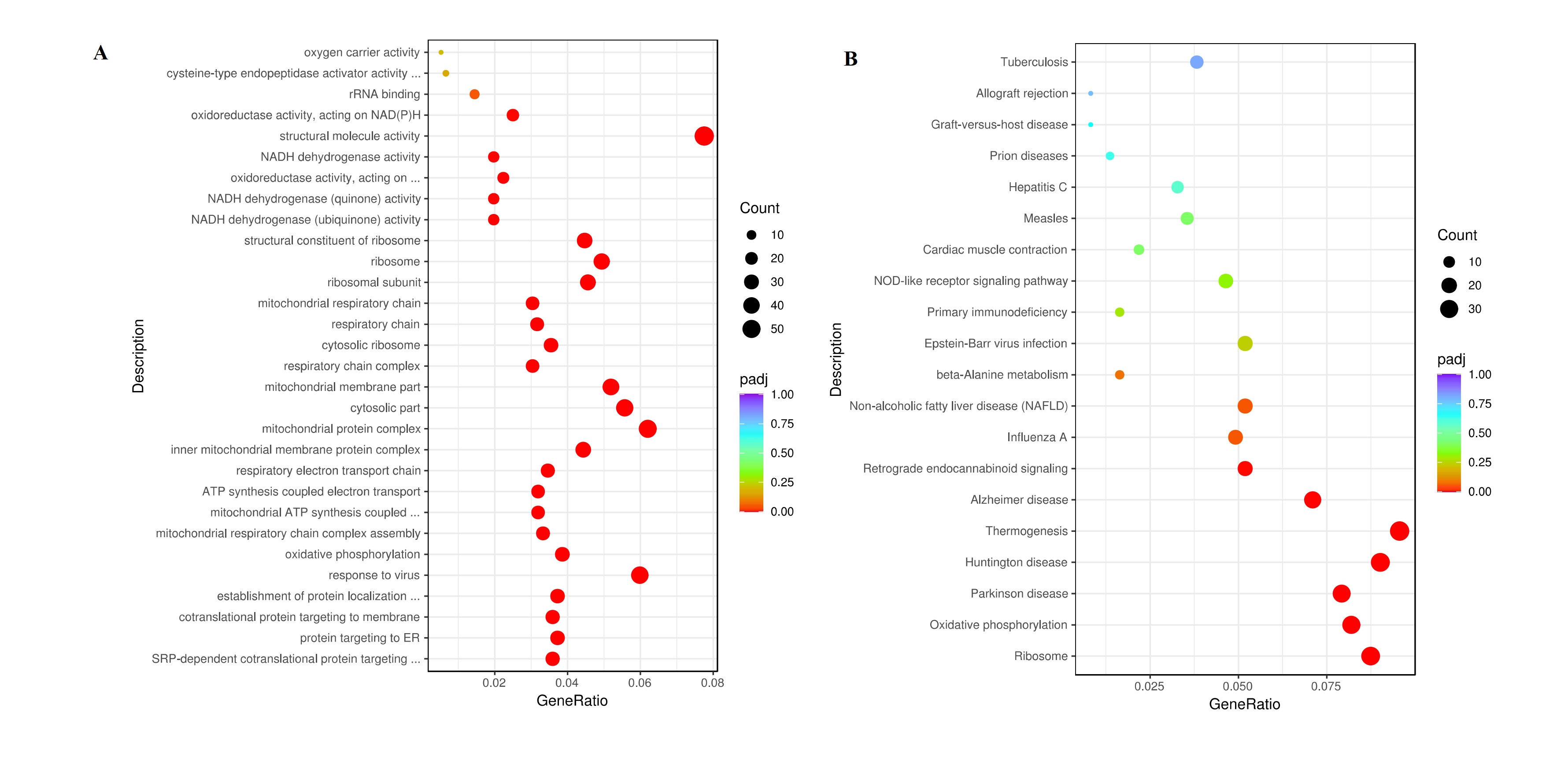

Supplement: Supplementary file 1 — Additional file 1. Figure S1: GO and KEGG scatter plots. A The top 30 GO terms with significant difference. B The top 20 KEGG terms with significant difference. [file 13023_2023_2705_MOESM1_ESM.tiff]

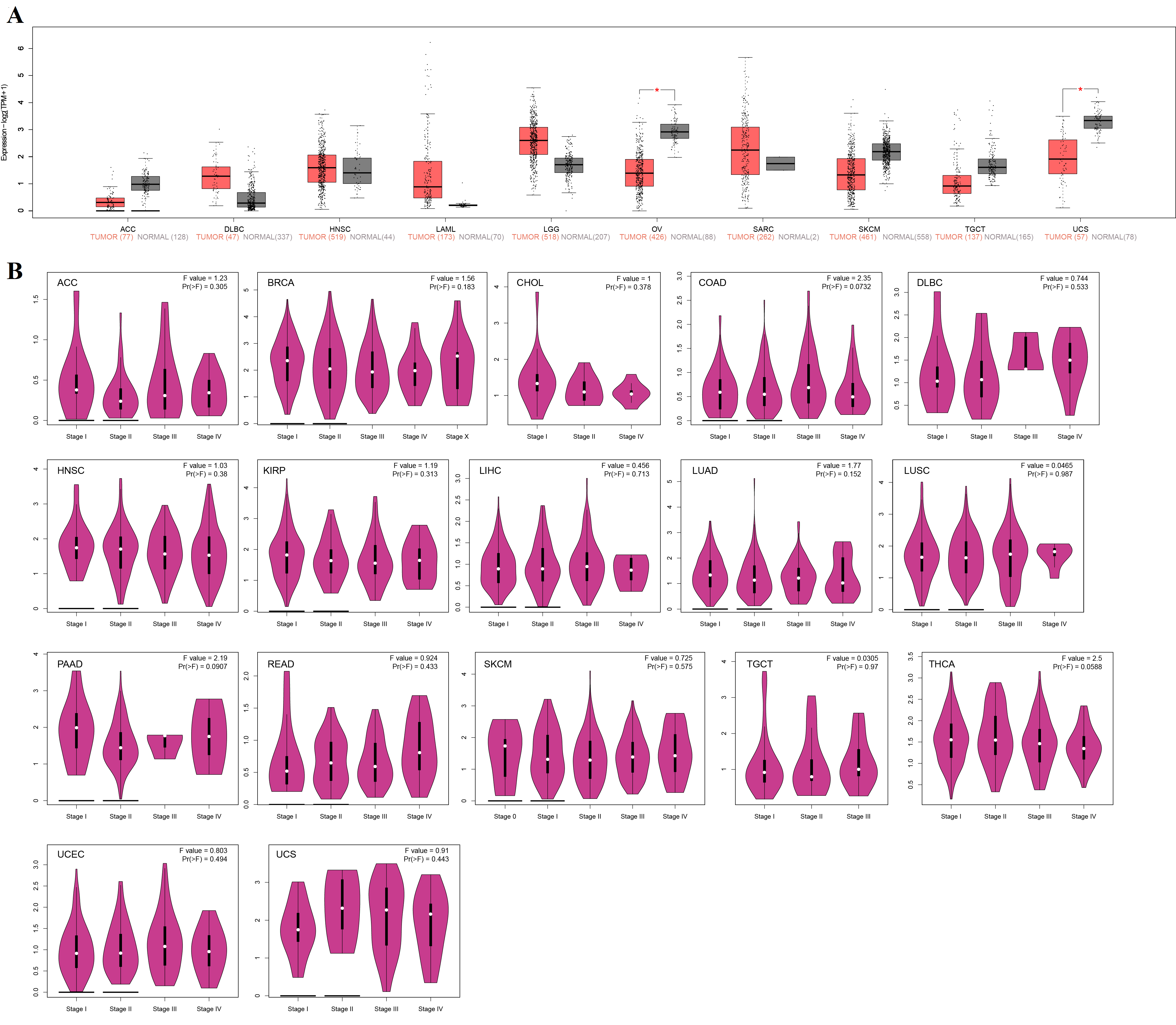

Supplement: Supplementary file 2 — Additional file 2. Figure S2: The expression of SETBP1 gene in tumor tissues and different tumor stages. A The expression of SETBP1 gene in tumor tissues. B The expressions of SETBP1 gene in different tumor stages. [file 13023_2023_2705_MOESM2_ESM.tiff]

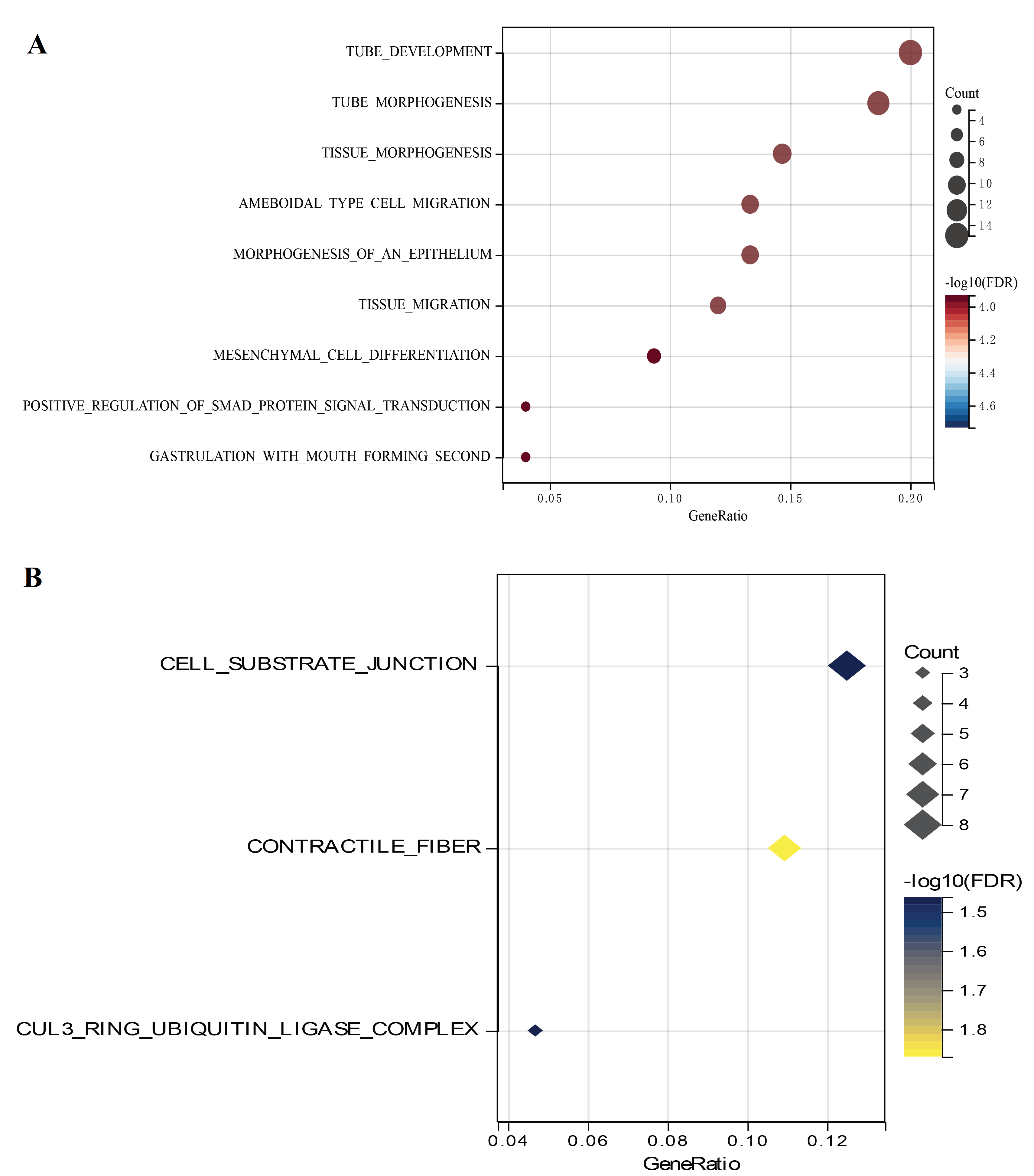

Supplement: Supplementary file 3 — Additional file 3. Figure S3: The enriched GO terms with significant difference. A SETBP1-related genes associated with the cellular component with significant difference. B SETBP1-related genes associated with the biological process with significant difference. [file 13023_2023_2705_MOESM3_ESM.tiff]
