# Supplementary material for: Identification of a novel de novo mutation of SETBP1 and new findings of SETBP1 in tumorgenesis
Source: Orphanet J Rare Dis. 2023 May 7;18:107. doi: 10.1186/s13023-023-02705-6 (PMC10165755; doi:10.1186/s13023-023-02705-6)
Supplement: Supplementary file 4 — Additional file 4. Table S1: The abbreviation and full name of the tumors. [file 13023_2023_2705_MOESM4_ESM.docx]

Table S1. The abbreviation and full name of the tumors.

| Abbreviation | Full name | Abbreviation | Full name |
| --- | --- | --- | --- |
| ACC | Adrenocortical carcinoma | LUAD | Lung adenocarcinoma |
| BLCA | Bladder Urothelial Carcinoma | LUSC | Lung squamous cell carcinoma |
| BRCA | Breast invasive carcinoma | MESO | Mesothelioma |
| CESC | Cervical squamous cell carcinoma and endocervical adenocarcinoma | OV | Ovarian serous cystadenocarcinoma |
| CHOL | Cholangio carcinoma | PAAD | Pancreatic adenocarcinoma |
| COAD | Colon adenocarcinoma | PCPG | Pheochromocytoma and Paraganglioma |
| COADREAD | Colon adenocarcinoma/Rectum adenocarcinoma Esophageal carcinoma | PRAD | Prostate adenocarcinoma |
| DLBC | Lymphoid Neoplasm Diffuse Large B-cell Lymphoma | READ | Rectum adenocarcinoma |
| ESCA | Esophageal carcinoma | SARC | Sarcoma |
| GBM | Glioblastoma multiforme | SKCM | Skin Cutaneous Melanoma |
| GBMLGG | Glioma | STAD | Stomach adenocarcinoma |
| HNSC | Head and Neck squamous cell carcinoma | STES | Stomach and Esophageal carcinoma |
| KICH | Kidney Chromophobe | TGCT | Testicular Germ Cell Tumors |
| KIPAN | Pan-kidney cohort (KICH+KIRC+KIRP) | THCA | Thyroid carcinoma |
| KIRC | Kidney renal clear cell carcinoma | THYM | Thymoma |
| KIRP | Kidney renal papillary cell carcinoma | UCEC | Uterine Corpus Endometrial Carcinoma |
| LAML | Acute Myeloid Leukemia | UCS | Uterine Carcinosarcoma |
| LGG | Brain Lower Grade Glioma | UVM | Uveal Melanoma |
| LIHC | Liver hepatocellular carcinoma |  |  |
